# The therapeutic benefit of upgrade to cardiac resynchronization therapy in patients with pacing-induced cardiomyopathy

**DOI:** 10.1016/j.hroo.2023.01.004

**Published:** 2023-01-25

**Authors:** Robert N. Kerley, Claire O’Dowling, Filipa Campos, Robbie D. Murphy, Katie A. Walsh, Gerard J. Fahy

**Affiliations:** ∗ Department of Cardiology, Cork University Hospital, Wilton, Cork, Ireland; † Department of Medicine, University College Cork, Cork, Ireland

**Keywords:** Pacing-induced cardiomyopathy, Cardiac resynchronization therapy, Heart failure, Pacing complications

## Abstract

**Background:**

Pacing-induced cardiomyopathy (PICM) is an important cause of heart failure in patients with a right ventricular pacing burden. Recent evidence suggests that an upgrade to cardiac resynchronization therapy (CRT) may confer benefit in PICM.

**Objective:**

To assess the extent and identify predictors of improvement following upgrade to CRT in patients with PICM.

**Methods:**

We retrospectively analyzed 43 patients undergoing CRT upgrade for PICM over the 10-year period of 2011 to 2021 at our center. All patients with PICM who underwent device upgrade from a dual- or single-chamber ventricular pacemaker to CRT were included. PICM was defined as a decrease of ≥10% in left ventricular ejection fraction (LVEF), resulting in an LVEF <50% among patients with ≥20% Right ventricular pacing burden without an alternative cause for cardiomyopathy.

**Results:**

LVEF significantly improved from 28.7% preupgrade to 44.3% post–CRT upgrade (*P <* .01). Of 37 patients with severe LV dysfunction, 34 (91.9%) improved to an LVEF >35% and 13 (35.1%) improved to an LVEF >50%. The LV end-diastolic diameter decreased from 5.9 cm preupgrade to 5.4 cm postupgrade (*P <* .01). Using linear regression, angiotensin-converting enzyme inhibitor or angiotensin receptor blocker use was associated with significant LVEF improvement (+7.21%, *P =* .05). We observed a low rate of complications, and 1 in 4 CRT upgrades required venoplasty (n = 10 of 43, 23.3%).

**Conclusion:**

We provide further evidence for the benefit of CRT upgrade in the management of patients with PICM.

**Key Findings**

▪ Pacing-induced cardiomyopathy (PICM) is an important cause of heart failure in patients with chronic right ventricular pacing.
▪ Recent evidence suggests that an upgrade to cardiac resynchronization therapy may reverse PICM.
▪ Our study shows, attenuation of electromechanical dyssynchrony through upgrade to a cardiac resynchronization therapy device can lead to near complete resolution of right ventricular PICM in 9 of 10 cases.
▪ Nine of 10 cases of severe PICM improved to a left ventricular ejection fraction >35% with a median of 9 months, avoiding the need for implantable cardioverter-defibrillator.

## Introduction

Conventional right ventricular pacing (RVP) is the most widely used method to treat symptomatic bradycardia and high-degree atrioventricular block.^1^, ^2^, ^3^ RVP causes electromechanical ventricular dyssynchrony, which in some patients may eventually result in left ventricular systolic dysfunction (LVSD).^3^, ^4^, ^5^ Definitions have varied across the literature but the most common criteria for pacing-induced cardiomyopathy (PICM) are (1) LV ejection fraction (LVEF) ≥50% before pacemaker implantation, (2) new onset of LVSD in patients with an RVP percentage ≥20% with an LVEF ≤50%, and (3) absence of alternative causes of LVSD.^6^, ^7^, ^8^ PICM is an important and increasingly recognized cause of heart failure in patients exposed to frequent RVP.^6^ Placement of an LV lead for biventricular pacing or cardiac resynchronization therapy (CRT) has been empirically recommended in international guidelines for the treatment of PICM despite limited clinical outcome data.^9^ A recent meta-analysis of patients with PICM treated with upgrade to CRT demonstrated an increase in LVEF of 10.9% and reduction in symptoms by 1 New York Heart Association (NYHA) functional class.^2^

In this study, we analyzed a large single-center cohort of consecutive CRT upgrades for patients with PICM over 10 years and sought to characterize the time course and degree of improvement in LVEF.

## Methods

### Study population

We performed a retrospective analysis of patients who underwent CRT upgrade for patients who developed a PICM at Cork University Hospital from January 2011 to December 2021. PICM was defined as a ≥10% decrease in LVEF with >20% RVP burden with a prior documented LVEF ≥50% either prior to or shortly after pacemaker implantation. The onset of PICM was considered the date of the first echocardiogram documenting LV dysfunction. A severe PICM was defined as an LVEF <35%. Patients with PICM were included in the study if they underwent an upgrade to a single- or dual-chamber biventricular pacemaker or implantable cardioverter-defibrillator (ICD) and had undergone repeat echocardiogram following device upgrade. Patients were excluded if they had an alternative cause of myocardial dysfunction such as myocardial infarction, valvular heart disease, tachycardia mediated cardiomyopathy, frequent premature ventricular contractions and/or uncontrolled hypertension. The study was approved by our local clinical research ethics committee. Written consent was not obtained, as the data were collected retrospectively and anonymized. The research in this study was conducted according to the Helsinki Declaration guidelines on human research.

### Clinical, echocardiographic, and electrophysiological parameters

Clinical and echocardiographic data were extracted from the hospital electronic and paper chart medical records and a separate database of implantable cardiac devices. Echocardiographic parameters were taken from the physiologist and reviewing cardiologist report. The preupgrade RVP percentage was obtained from the interrogation uploaded to this database immediately preceding upgrade. Preupgrade unpaced QRS duration and morphology were measured manually from the electrocardiography preceding upgrade, which demonstrated conduction complexes. Preupgrade paced QRS duration was measured from the electrocardiography immediately preceding CRT upgrade. A CRT response was defined as an improvement in LVEF ≥5%. Transthoracic echocardiography postupgrade was performed at 3 to 6 months postupgrade. Ventricular arrhythmia was defined as sustained if longer than 30 seconds or requiring ICD therapy.

### Statistical analysis

Variables are presented as the mean ± SD if continuous or number and percentage if categorical. To compare mean preupgrade LVEF with mean LVEF postupgrade, Student’s paired *t* test was used for statistical analysis. To determine predictors of LVEF improvement following CRT upgrade, multivariate linear regression analysis was performed. All reported *P* values are 2-tailed, with *P* values <.05 considered statistically significant. Analyses were performed using the software package SPSS Statistics 24 (IBM, Armonk, NY).

## Results

### Demographics

**Figure 1:**
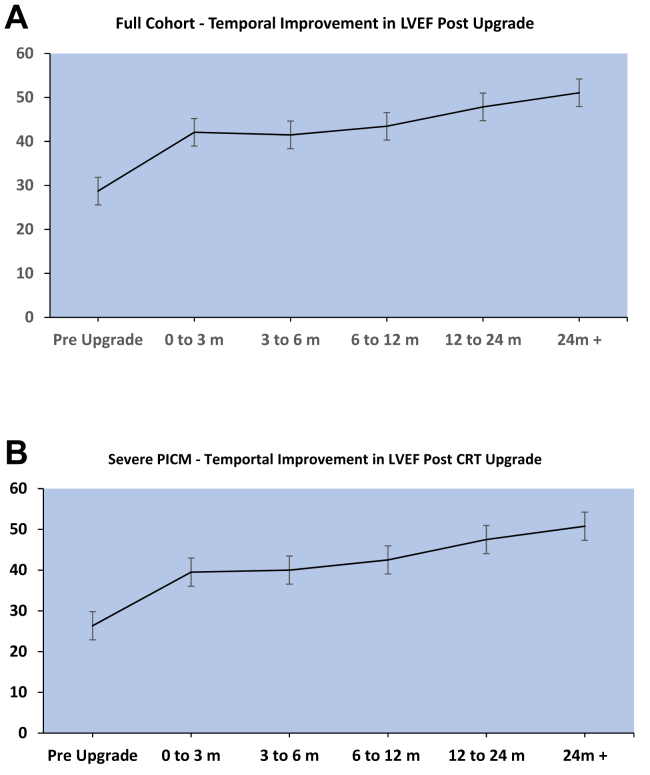
Left ventricular ejection fraction (LVEF) temporal response to cardiac resynchronization therapy (CRT) upgrade for the full cohort **(A)** and those with severe pacing induced cardiomyopathy **(B)**. m = month; PICM = pacing-induced cardiomyopathy.

**Figure 2:**
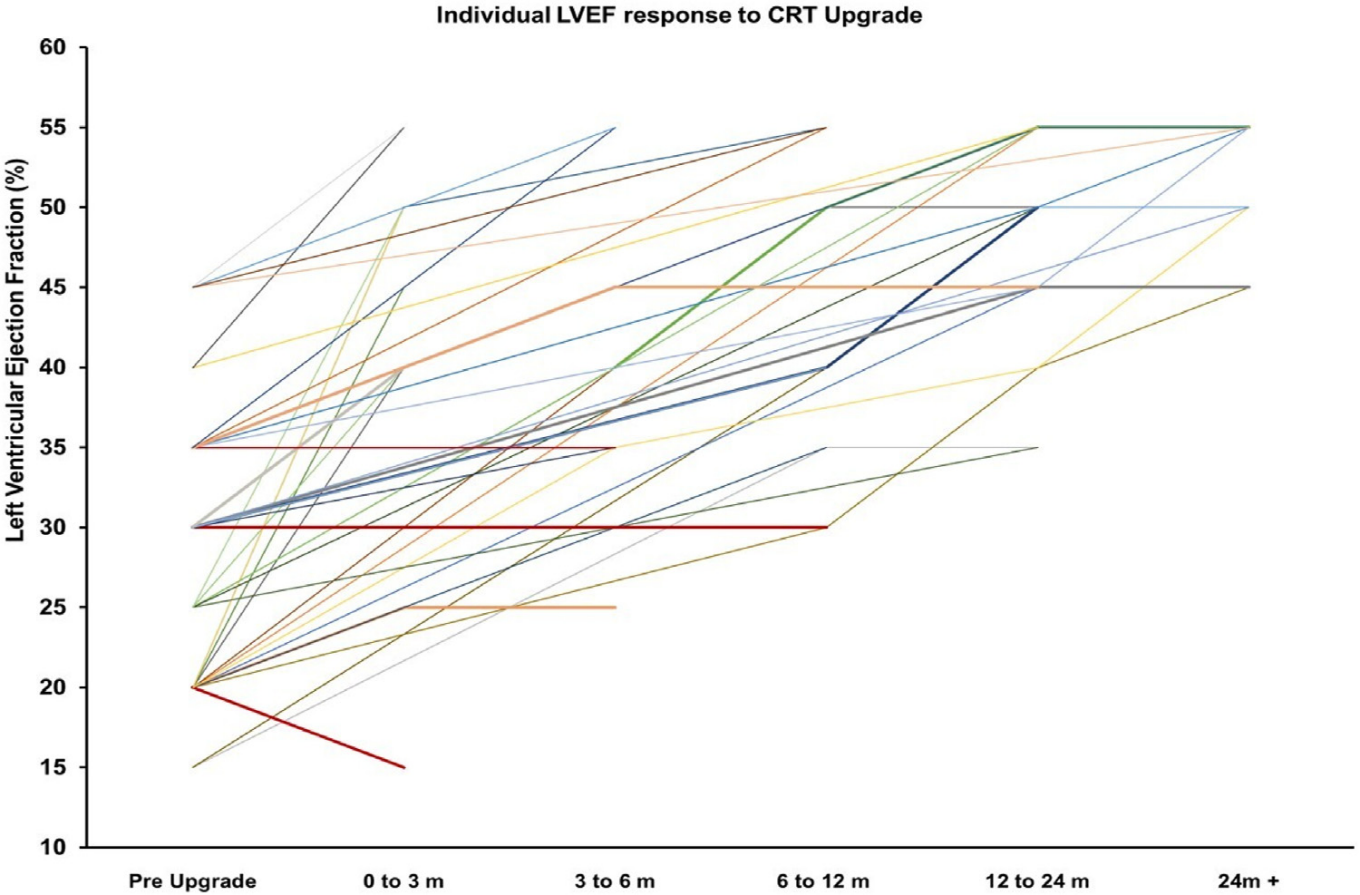
Individual temporal response of left ventricular ejection fraction (LVEF) to cardiac resynchronization therapy (CRT) upgrade. Nonresponders have been highlighted in red. m = month.

Over a 10-year period at our hospital, 43 patients underwent device upgrade for PICM. Baseline characteristics for these patients are outlined in Table 1. In 43 patients undergoing upgrade, LVEF significantly improved from 28.7% preupgrade to 44.3% post–CRT upgrade (*P <* .01) (Table 2). A median of 2 echocardiograms were performed over a median of 7.3 months of follow-up (Figures 1 and 2). A total of 38 (88.4%) patients had an LVEF improvement of >5%, 25 (58.1%) patients had an improvement of >10%. LV end-diastolic dimension (LVIDd) decreased from 5.9 cm preupgrade to 5.4 cm postupgrade (*P <* .001). Of 37 patients with severe LV dysfunction, 34 (91.9%) improved to an LVEF >35% with a median time of 9.4 months, and 13 (35.1%) improved to an LVEF >50% with a median time of 14.0 months. LVIDd improved from 6.2 cm to 5.6 cm postupgrade in severe PICM (*P <* .001). Ten patients with severe PICM had undergone initial upgrade to CRT defibrillator, with no patients requiring ICD lead implantation postupgrade. Of 3 patients with severe PICM who did not improve to an LVEF >35%, 2 patients were initially upgraded to CRT defibrillator and 1 died 4 months post–device implantation from respiratory sepsis. Of 6 patients with mild-to-moderate PICM, postupgrade LVEF improved from 43.3% to 55.8% (*P <* .01) and LVIDd remained unchanged from 4.65 cm to 4.68 cm (*P =* .94). Most LVEF improvement occurred within the first 3 months, with a mean LVEF improving from 28.7% to 41.5%. We observed an average reduction in NYHA functional class from 2.39 preupgrade to 1.33 postupgrade for all patients with PICM and 2.40 preupgrade to 1.33 postupgrade in patients with severe PICM. There was no change in NYHA functional class in patients with mild to moderate PICM (2.3 vs 1.3, *P =* .051). Of the 5 patients that did not respond to CRT, all were men, RVP percentage was 93.4 ± 13.2%, and paced QRS was 194.3 ± 21.6 ms preupgrade and 151.8 ± 35.5 ms postupgrade (Supplemental Table 1). Supplemental Figure 1 shows an example of the typical change in LVEF, QRS, and mitral valve Doppler inflow.

**Table 1:** Demographics of PICM patients

Demographics, comorbidities and medications at upgrade	
Female	13 (30.2)
Age, y	77.0 ± 10.8
Coronary artery disease	21 (48.8)
Atrial fibrillation	22 (51.2)
Clinical heart failure	38 (88.4)
Beta-blocker use	35 (81.4)
ACE inhibitor or ARB use	31 (72.1)
MRA use	16 (37.2)
Loop diuretic use	28 (65.1)
Native QRS	119.2 ± 34.6
LBBB	5 (11.6)
RBBB	5 (11.6)
Pacemaker indication	
Sinus node dysfunction	5 (11.6)
Atrioventricular block	38 (88.4)
Right ventricular pacing percentage	89.5 ± 22.5
Time to PICM, mo	97.2 ± 96.1
Time from PICM to upgrade, mo	3.2 ± 3.5
Need for venuloplasty during upgrade	10 (23.3)
CRT defibrillator insertion	11 (25.6)

Values are n (%) or mean ± SD.

ACE = angiotensin-converting enzyme; ARB = angiotensin receptor blocker; CRT = cardiac resynchronization therapy; LBBB = left bundle branch block; MRA = mineralocorticoid receptor antagonist; PICM = pacing-induced cardiomyopathy; RBBB = right bundle branch block.

**Table 2:** Comparison of patients with PICM pre- and postupgrade to CRT

	Preupgrade	Postupgrade	*P* Value
Full cohort (**N** = 43)			
Paced QRS, ms	186.1 ± 23.3	158.1 ± 25.9	.022
Left ventricular end-diastolic diameter, cm	5.9 ± 0.9	5.4 ± 0.9	.003
Left ventricular ejection fraction, %	28.7 ± 8.4	44.3 ± 9.4	<.001
NYHA functional class	2.4 ± 0.5	1.3 ± 0.5	<.001
Severe PICM (n = 37)			
Paced QRS, ms	186.3 ± 21.2	163.5 ±19.2	<.001
Left ventricular end-diastolic diameter, cm	6.2 ± 0.8	5.6 ± 0.9	.006
Left ventricular ejection fraction, %	26.4 ± 6.5	42.6 ± 9.2	<.001
NYHA functional class	2.4 ± 0.5	1.3 ± 0.5	<.001
Mild-to-moderate PICM (n = 6)			
Paced QRS, ms	176.0 ± 36.1	139.8 ± 43.7	.031
Left ventricular end-diastolic diameter, cm	4.7 ± 0.5	4.7 ± 0.5	.946
Left ventricular ejection fraction, %	43.3 ± 2.6	55.8 ± 2.04	<.001
NYHA functional class	2.3 ± 0.6	1.3 ± 0.6	.051

Values are mean ± SD.

NYHA = New York Heart Association; PICM = pacing-induced cardiomyopathy.

### Ventricular arrhythmia

Among the entire cohort, 3 (7.0%) patients had a sustained ventricular arrythmia following upgrade implantation with ventricular rates of 150 to 182 beats/min. Of those patients, 2 were treated with antitachycardia pacing and 1 was treated with a single shock. All had a severe PICM, all had an initial CRT defibrillator implant, and all responded to CRT. Two patients had a normal LVEF, and 1 had an LVEF 45% to 50% at the time of arrythmia. All events occurred within 18 months of follow-up. Among the entire cohort, 15 (34.9%) patients had nonsustained ventricular tachycardia within 18 months of follow-up.

### Predictors of LVEF improvement

Using univariable regression analysis, among the 43 patients with PICM, age, lower preupgrade LVEF, angiotensin-converting enzyme (ACE) inhibitor or angiotensin receptor blocker (ARB) prescription, and mineralocorticoid receptor antagonist prescription were associated with LVEF improvement (Table 3). In multivariable regression analysis, only ACE inhibitor or ARB prescription (+7.21%, 95% confidence interval –0.10% to 14.53%, *P* = .05) remained significantly associated with LVEF improvement postupgrade (Table 3). In a separate univariate analysis of 37 patients with severe PICM, ACE inhibitor or ARB use and shorter preupgrade paced QRS were associated with an improvement in LVEF to >35%. In a multivariate analysis, ACE inhibitor or ARB prescription and preupgrade paced QRS were not significantly associated with an LVEF improvement to greater than 35% (Table 4).

**Table 3:** Factors associated with improvement in LVEF postupgrade

	Univariate			Multivariate		
	LVEF increase (%)	95% CI (%)	*P* Value	LVEF increase (%)	95% CI (%)	*P* Value
Female	0.30	–5.76 to 6.29	.93			
Age	–0.22	–0.47 to 0.03	.08	–0.18	–0.43 to 0.08	.18
Coronary artery disease	–0.21	–5.74 to 5.33	.94			
Atrial fibrillation	0.67	–4.86 to 6.20	.81			
Clinical heart failure	7.45	–0.86 to 15.76	.08			
Beta-blocker use	0.71	–6.39 to 7.82	.84			
ACE inhibitor or ARB use	7.16	1.42 to 12.91	.02	7.21	–0.10 to 14.53	.05
MRA use	7.54	2.33 to 12.74	.01	4.51	–1.57 to 10.59	.14
Loop diuretic use	0.89	–4.91 to 6.69	.76			
Pacemaker indication—complete heart block	–3.87	–12.42 to 4.68	.37			
CRT defibrillator implantation	3.49	–2.75 to 9.74	.27			
Right ventricular pacing percentage	–0.05	–0.08 to 0.17	.46			
LBBB	–1.64	–9.61 to 6.33	.68			
RBBB	7.63	–1.59 to 16.85	.10			
Time to PICM	–0.01	–0.04 to 0.02	.64			
Time from PICM to upgrade	–0.43	–1.22 to 0.36	.27			
Need for venuloplasty during upgrade	2.50	–4.00 to 9.00	.44			
Native QRS	0.01	–0.14 to 0.16	.90			
Preupgrade paced QRS	0.06	–0.05 to 0.18	.26			
Postupgrade paced QRS	–0.06	–0.17 to 0.06	.30			
Change in QRS postupgrade	–0.06	–0.18 to 0.05	.28			
Preupgrade EF	–0.42	–0.72 to –0.11	.01	–0.26	–0.65 to 0.13	0.18
Preupgrade LVIDd	2.31	–1.15 to 5.77	.18			
RV septal lead placement	2.71	0.23 to 32.34	.43			

ACE = angiotensin-converting enzyme; ARB = angiotensin receptor blocker; CI = confidence interval; CRT = cardiac resynchronization therapy; EF = ejection fraction; LBBB = left bundle branch block; LVEF = left ventricular ejection fraction; LVIDd = left ventricular end-diastolic diameter; MRA = mineralocorticoid receptor antagonist; PICM = pacing-induced cardiomyopathy; RBBB = right bundle branch block; RV = right ventricular.

**Table 4:** Factors associated with an LVEF improvement to >35% in patients with severe PICM

	Univariate			Multivariate		
	Odds ratio	95% CI	*P* Value	Odds ratio	95% CI	*P* Value
Female	1.57	0.15–15.97	.71			
Age	1.03	0.96–1.12	.38			
Coronary artery disease	0.71	0.10–4.86	.73			
Atrial fibrillation	5.54	0.55–55.49	.15			
Clinical heart failure	3.63	0.26–49.70	.34			
Beta-blocker use	0.92	0.09–9.82	.95			
ACE inhibitor or ARB use	6.50	0.88–27.90	.07	6.69	0.42–106.26	.18
MRA use	1.17	0.17–7.96	.88			
Loop diuretic use	0.48	0.05–4.81	.53			
Pacemaker indication	0.74	0.07–8.09	.81			
CRT defibrillator implantation	0.50	0.07–3.55	.49			
RV pacing percentage	1.02	0.99–1.06	.24			
LBBB	0.41	0.03–5.01	.49			
RBBB	1.35	0.12–14.73	.81			
Time to PICM	1.01	0.99–1.02	.46			
Time from PICM to upgrade	0.88	0.70–1.10	.25			
Need for venuloplasty during upgrade	1.39	0.14–14.36	.78			
Native QRS	1.02	0.97–1.07	.42			
Preupgrade paced QRS	1.09	0.99–1.20	.09	1.12	0.97–1.29	.13
Postupgrade paced QRS	1.00	0.95–1.04	.86			
Preupgrade EF	1.04	0.89–1.21	.62			
Preupgrade LVIDd	0.64	0.17–2.32	.49			
RV septal lead placement	0.97	0.14–6.67	.98			

ACE = angiotensin-converting enzyme; ARB = angiotensin receptor blocker; CI = confidence interval; CRT = cardiac resynchronization therapy; EF = ejection fraction; LBBB = left bundle branch block; LVIDd = left ventricular end-diastolic diameter; MRA = mineralocorticoid receptor antagonist; PICM = pacing-induced cardiomyopathy; RBBB = right bundle branch block; RV = right ventricular.

### Complications

We observed a low complication rate among patients undergoing CRT implantation. Among 43 patient there was 1 device infection requiring device extraction, 2 hematomas, and 1 left basilic vein deep vein thrombosis. In terms of mortality, there was 1 death within 12 months of upgrade implantation, which was caused by respiratory sepsis.

## Discussion

Our findings from a 10-year single-center experience showed that (1) upgrade to CRT in patients with PICM resulted in a mean increase in LVEF from 28.7% to 44.3%; (2) among patients with severe PICM defined as an LVEF <35%, 91.9% improved to LVEF >35% and 35.1% improved to an LVEF >50%; (3) rates of ventricular arrythmia were low, with 3 sustained arrhythmias within the first 18 months of upgrade; and (4) those prescribed ACE inhibitors or ARBs observed the greatest improvement in LVEF.

### Response to CRT upgrade

Our study adds to the existing literature that demonstrates the beneficial effects of upgrade to CRT in patients with PICM. We observed a 92% CRT response rate, which is similar to previous cohort studies.^6^^,^^10^ A recent meta-analysis including 4 studies with PICM showed a response rate of 76% to 85.7%.^2^ Our study observed an LVEF improvement of 14%, which is in keeping with previous reports of 14% to 19%.^6^^,^^11^^,^^12^ Nazeri and colleagues^13^ observed a smaller increase of 6.2% and a lower response rate at 76%. The smaller improvement may be due to inclusion of patients with concomitant conditions such as ischemic cardiomyopathy and infiltrative cardiomyopathy, which we excluded in our cohort. Equally, the study by Nazeri and colleagues included a smaller cohort of 21 patients and much lower RVP burden, which may have affected the results.

### Response in patients with severe PICM

Importantly, in patients with severe PICM, defined as an LVEF <35%, we observed an excellent response to CRT, with 9 of every 10 patients improving to an LVEF >35%. This is a similar finding to that observed by Khurshid and colleagues^6^ and Nazeri and colleagues^13^ in which 72% and 76% of patients with severe PICM improved to an LVEF >35%, respectively. Furthermore, the higher CRT response observed in our study may be due to the shorter time interval between diagnosis of PICM and upgrade to CRT of 3.5 months vs 18.0 months in previous studies.^6^ In our linear regression analysis, a wider preupgrade paced QRS duration was associated with increased magnitude of LVEF improvement. This suggests that attenuation of electromechanical dyssynchrony through upgrade to a CRT device can lead to near-complete resolution of RVP induced cardiomyopathy in 9 of 10 cases. In patients with atrioventricular block with pacing-dependent rhythm, regardless of the pacing site, the paced QRS duration has been shown to be a major determinant in the occurrence of PICM.^8^

### Predictors of LVEF Improvement

In univariate analysis, our study observed that patient age, preupgrade LVEF, and prescription of an ACE inhibitor or ARB and mineralocorticoid receptor antagonist were significant predictors of LVEF improvement. In multivariate analysis, only ACE inhibitor or ARB use remained marginally associated with LVEF improvement, and this did not reach statistical significance. LV mechanical dyssynchrony causes by chronic RVP have been shown to be diminished with an ACE inhibitor in an animal model of heart failure but have not been previously shown to be of benefit in clinical cohort studies, although data are admittedly limited.^14^ The mechanical dyssynchrony caused by chronic RVP is distinctly different to the neurohormonal mechanisms underpinning the improvement associated with renin-angiotensin-aldosterone system inhibition in other forms of systolic heart failure. This is the first study to observe a concomitant effect of ACE inhibitor or ARB use with CRT upgrade in the treatment of patients with PICM. Among patients with PICM resulting in LVEF >35%, predictors of improvement to LVEF >35% following CRT upgrade are of particular interest to guide the decision of whether to upgrade to a biventricular pacemaker or ICD.^10^ Interestingly, when comparing the response to CRT in patients with severe PICM vs patients with mild-to-moderate PICM, we observed a significant improvement in LVIDd in patients with severe PICM but not with mild-to-moderate PICM (Table 2). Both groups observed a significant improvement in LVEF, paced QRS duration, and NYHA functional class, but only patients with a severe PICM observed a change in LV dimension. This suggests that for patients with a mild-to-moderate PICM, the primary culprit is intraventricular dyssynchrony, whereas for patients with a severe PICM, there is a neurohormonal component that may account for the improvement observed with LV remodeling medications. Our study provides reasonable evidence that use of LV remodeling medications, specifically ACE inhibitors or ARBs, improves outcomes in patients with PICM.

### Timing of LVEF improvement and ventricular arrhythmia

The majority of LVEF recovery occurred within the first 3 months of follow-up. Our study had a time to upgrade of 3 months from diagnosis of PICM with a higher rate of response to CRT compared with other studies in the available literature (Table 5). This suggests that there may be a window of opportunity for patients with PICM, although this was not found to be statistically significant in our study. Furthermore, when compared with 4 other studies available in the literature, response rates of >85% have been observed for patients with a PICM diagnosis to upgrade time of up to 144 months. This supports the hypothesis that PICM is caused by electromechanical dyssynchrony, rather than by a structural heart disease such as myocardial fibrosis. This suggests that a robust improvement can be expected even late into the diagnosis of PICM. The vast majority of our patients received a CRT pacemaker device, none of which required upgrade to a CRT defibrillator postimplantation. Three patients had sustained ventricular arrhythmias at 3, 6, and 15 months, respectively, with 2 episodes terminated with antitachycardia pacing and the third with a single shock. This is in keeping with previous studies that have observed a low rate of sudden cardiac death in patients with a PICM.^6^^,^^15^

**Table 5:** Summary of previous studies published on PICM and response to CRT upgrade

Study	Design	n	Female (%)	RV pacing (%)	Time to upgrade (mo)	pQRS preupgrade (ms)	Follow-up (mo)	Response
Nazeri et al^13^	Case series	21	38.1	40.9 ± 13.2	3.8	159 ± 27	4.9	76
Khurshid et al^6^	Case series	69	37.7	95.7 ± 9.1	56.4	184 ± 21.7	7.7	85.5
Gwag et al^11^	Case cohort	7	42.9	99.6 ± 0.9	NR	185.5 ± 51.4	15.8	85.7
Schwerg et al^12^	Case series	20	46	99 ± 1	144	NR	36	85
Present study	Case series	43	30.2	89.5 ± 22.5	3.2	186.1 ± 23.3	9.4	88.4

CRT = cardiac resynchronization therapy; NP = not reported; pQRS = paced QRS; RV = right ventricular.

### The potential role of conduction system pacing

It is important to mention that while CRT has an established role in the management of PICM as outlined by the data in our study, future management strategies for PICM must include the possibility of conduction system pacing (CSP). Results of the recent Left Bundle Branch Pacing Versus Biventricular Pacing for Cardiac Resynchronisation Therapy trial comparing LBB pacing to CRT showed a statistically significant increase in LVEF with CSP.^16^ Placement of a His bundle or LBB area pacing lead may prove a more effective means of ventricular resynchronization and resolve the issue of electromechanical dyssynchrony, thought to cause PICM. At the time of this study’s publication, there have been no studies assessing the role of upgrade to a CSP device in the treatment of PICM.

### Limitations

Our findings should be interpreted within the limitations of the study design. First, this was a retrospective analysis; therefore, echocardiograms performed were not done based on a study protocol at specific intervals. Equally, echocardiograms were performed at different timing intervals, making it difficult to be exact in the timing of recovery or lack thereof. Second, while significant efforts were made to exclude other causes of reduced systolic LVEF, our cohort was not genotyped, and it is entirely plausible that many patients had concomitant structural heart disease. Third, due to the strict exclusion criteria, our results cannot be extrapolated to a group with concomitant structural heart disease. Presumably, the degree of LVEF improvement would be decreased in such patients, but we cannot make inferences based on our clinical cohort. Fourth, the average RVP percentage was 89% in our cohort, which limited our ability to assess the efficacy of CRT upgrade in patients with PICM with lower pacing percentages.

## Conclusion

Our study has shown a significant clinical and echocardiographic response to CRT in patients with a PICM. Previous randomized controlled trials have shown conflicting results regarding de novo implantation of CRT pacemaker devices over RVP.^17^^,^^18^ However, the robust response observed in our study does not argue for de novo CRT implantation in those with high RVP pacing burden (RVP burden >20%), as most patients do not develop PICM. Rather, our data show that those who do develop an appreciable decrease in LVEF >10% from previous in the presence of chronic RVP >20% would benefit from upgrade to a CRT device. Those that do not recover within 1 year of implantation of a CRT pacemaker should be considered for CRT defibrillator implantation. Our data support guideline recommendations for the use of LV remodeling medications in patients with clinical heart failure and reduced ejection fraction who develop PICM as a concomitant management strategy with CRT pacemaker upgrade. Future studies and management strategies may take advantage of CSP as an upgrade in PICM or avoid the issue entirely, but this has yet to be studied.^19^^,^^20^
