# Supplementary material for: The therapeutic benefit of upgrade to cardiac resynchronization therapy in patients with pacing-induced cardiomyopathy
Source: Heart Rhythm O2. 2023 Jan 25;4(4):225–31. doi: 10.1016/j.hroo.2023.01.004 (PMC10134389; doi:10.1016/j.hroo.2023.01.004)
Supplement: Supplementary Figure 1 [file mmc1.docx]

| 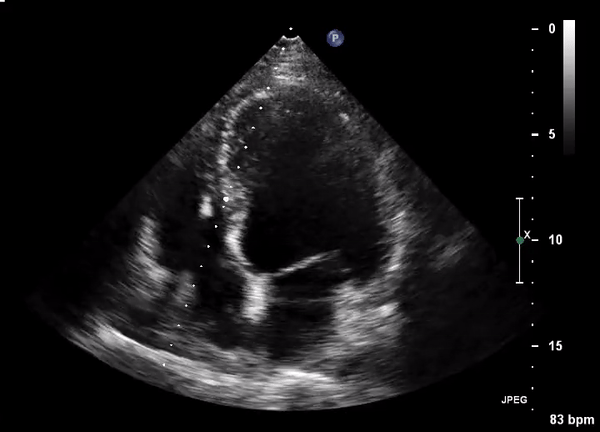  **(A)** | 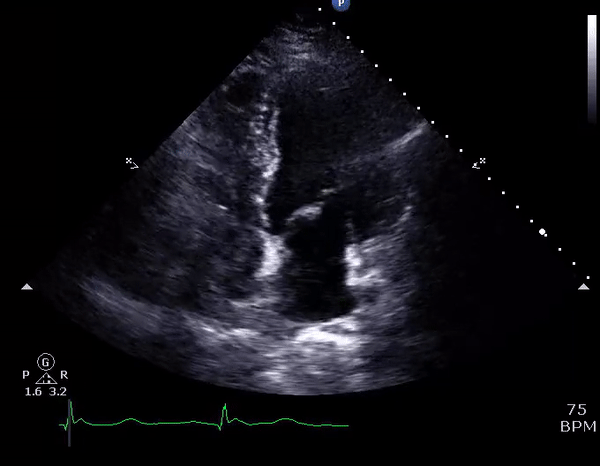 |
| --- | --- |
| 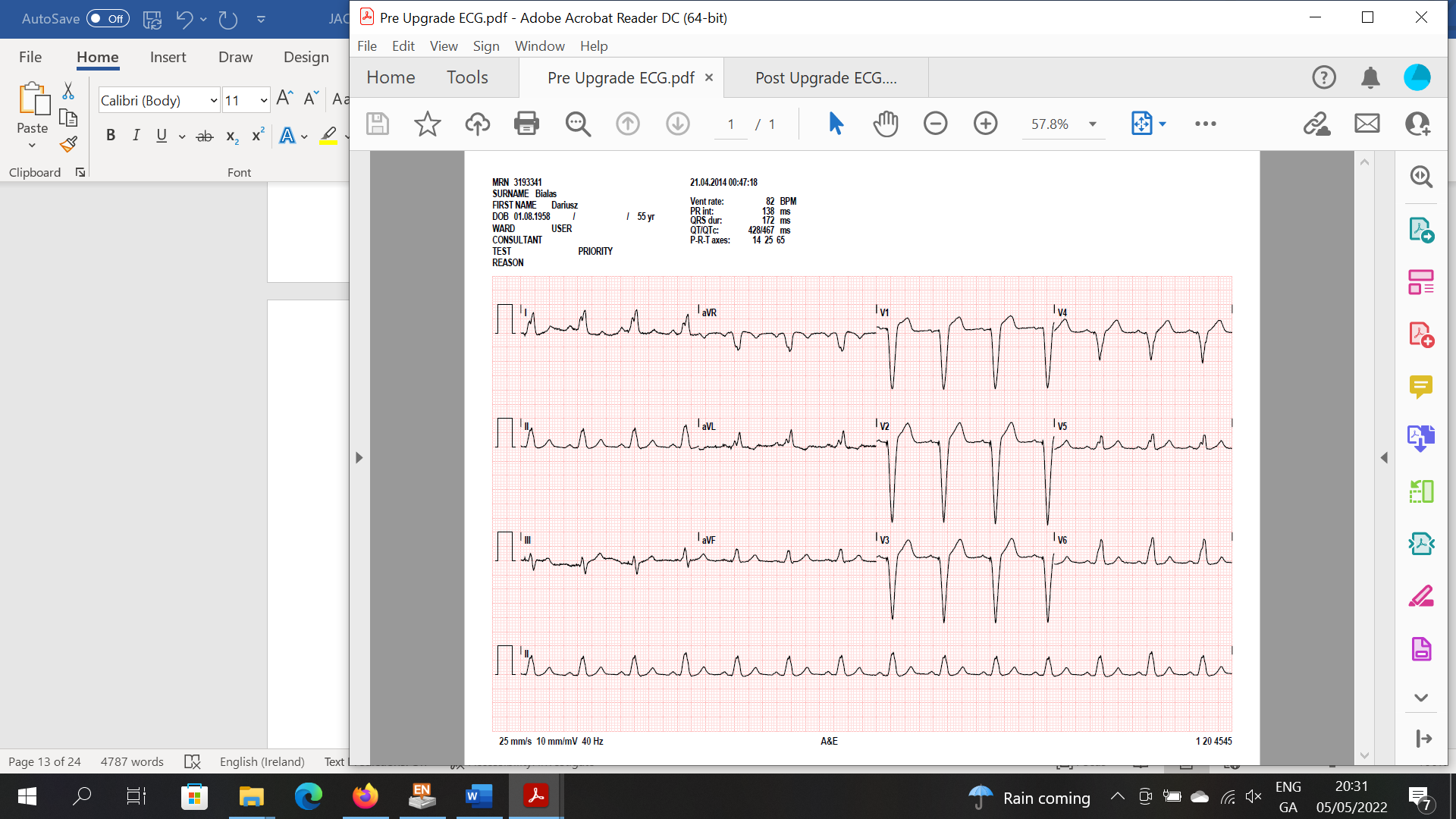  **(C)** | 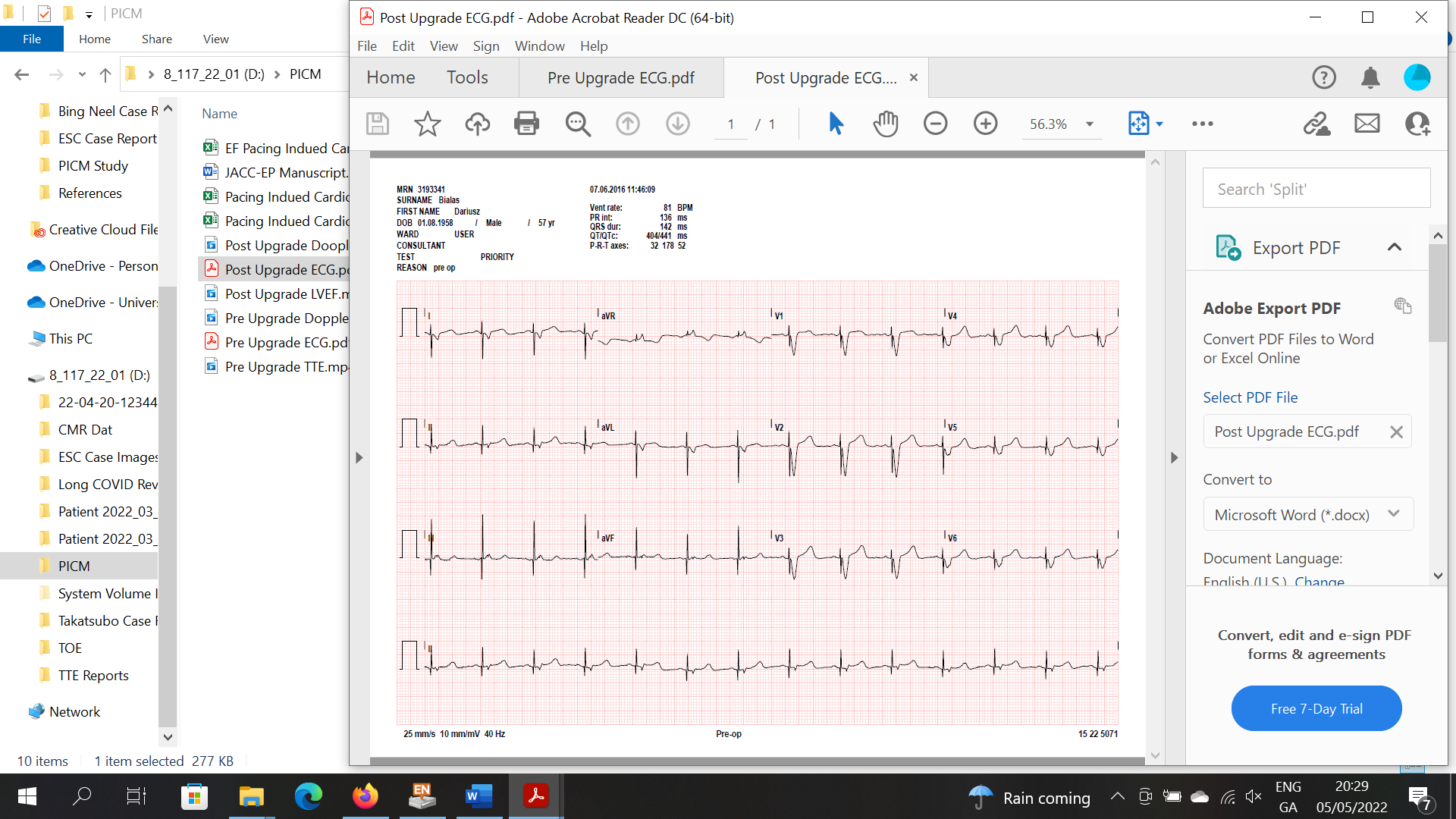  **(D)**  **(B)** |
| 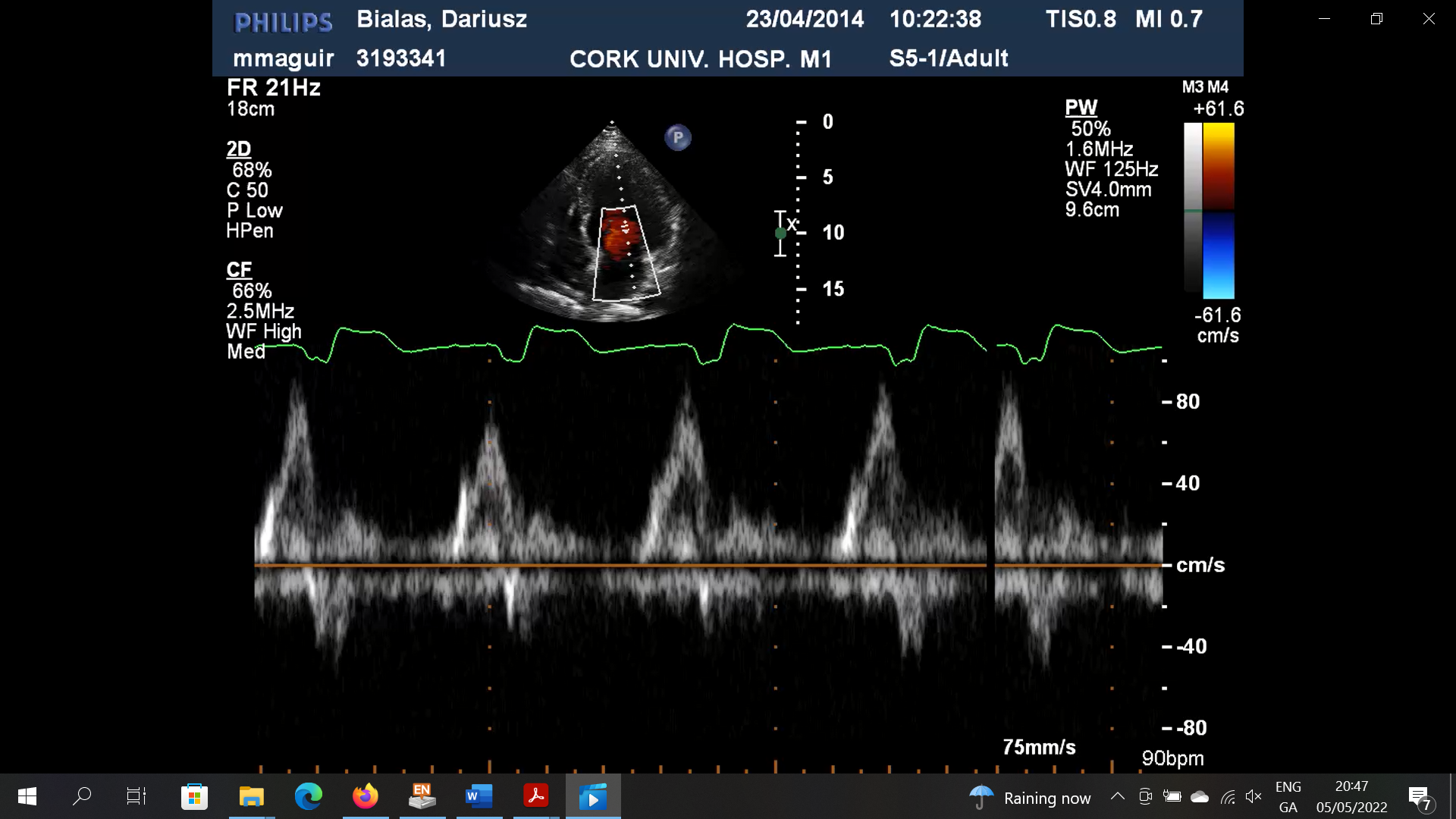 | 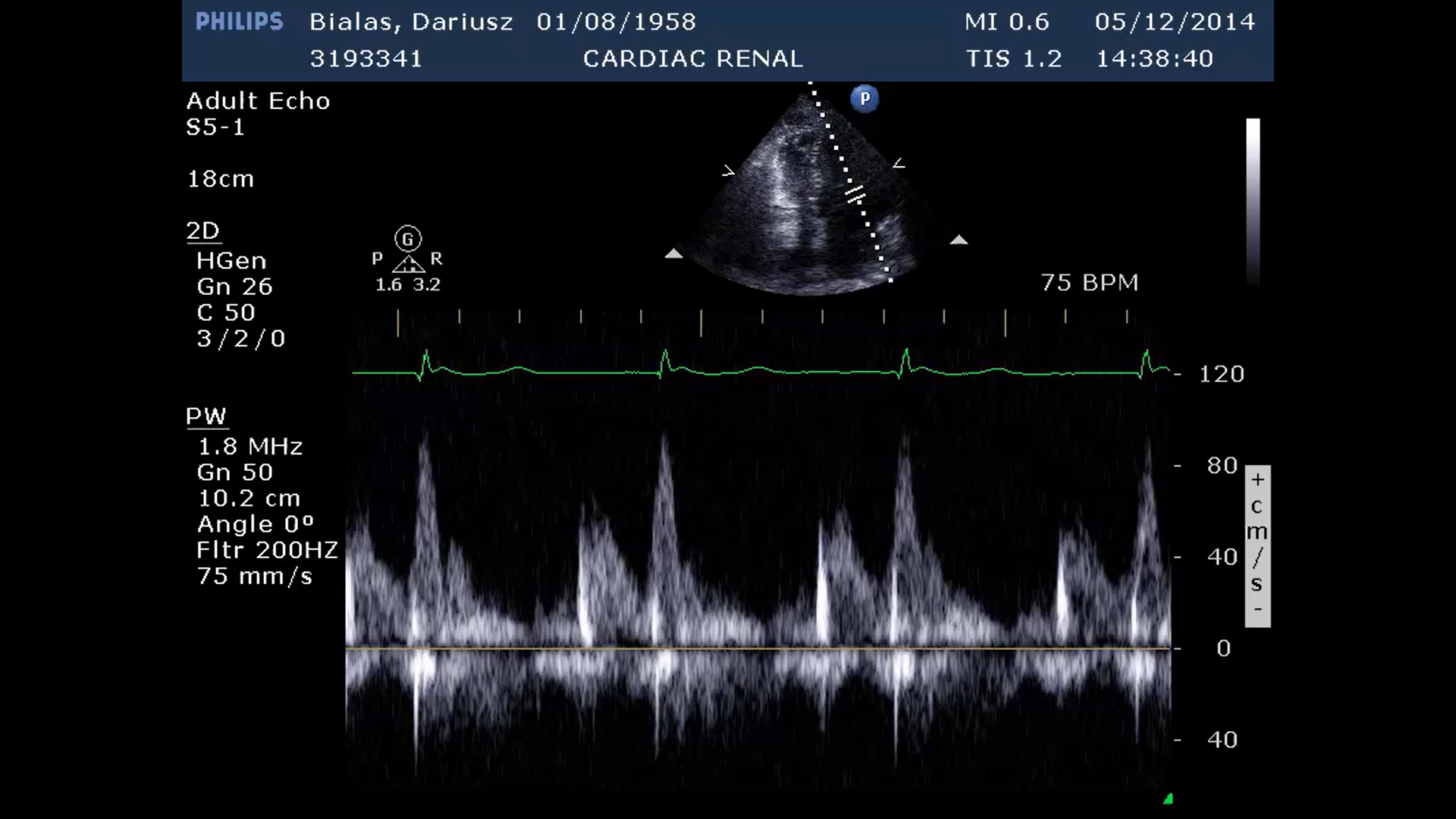  **(F)** |

**(E)**

**Supplementary Figure 1. The Effects of CRT Upgrade on LV systolic function paced QRS and diastolic function.**

Patient 23 is shown above who had a dual chamber pacemaker inserted two years prior to the development of a pacing induced cardiomyopathy. Pre- and post-upgrade cardiac resynchronisation upgrade images are shown for left ventricular ejection fraction (A & B), paced QRS (C & D) and mitral valve doppler inflow (E & F). LV function improved from 20% pre-upgrade (A) to 50% post-upgrade (B). Paced QRS decreased from 172 ms pre-upgrade (C) to 142 ms post upgrade (D). A decrease in mitral valve inflow E velocity from 79.5 cm/s to 68.4 cm/s and prolongation in E wave deceleration time from 130 ms to 240 ms was observed post upgrade indicating an improvement in diastolic left ventricular function.
