# Supplementary material for: The therapeutic benefit of upgrade to cardiac resynchronization therapy in patients with pacing-induced cardiomyopathy
Source: Heart Rhythm O2. 2023 Jan 25;4(4):225–31. doi: 10.1016/j.hroo.2023.01.004 (PMC10134389; doi:10.1016/j.hroo.2023.01.004)
Supplement: Supplementary Table 1 [file mmc2.docx]

**Supplementary Table 1 Demographics of Cardiac Resynchronisation Non-Responders.**

| Patient | Age | Gender | RV pacing | Ejection Fraction | | Paced QRS | | LV position | Time of echo follow up | LVIDd pre-upgrade | BiV Pacing % |
| --- | --- | --- | --- | --- | --- | --- | --- | --- | --- | --- | --- |
|  |  |  |  | Pre-upgrade | Post-upgrade | Pre-upgrade | Post-upgrade |  |  |  |  |
| 1 | 88 | Male | 100 | 20.00 | 15 | 221 |  | Lateral | 4.6 | 5.2 | 100 |
| 2 | 87 | Male | 70 | 30 | 30 | 170 | 157 | Lateral | 2.3 | 6.1 | 100 |
| 3 | 74 | Male | 99 | 35 | 35 | 200 | 200 | Lateral | 9.7 | 5.3 | 92 |
| 4 | 75 | Male | 98 | 20 | 25 |  | 125 | Lateral | 10.0 | 6.0 | 98.4 |
| 5 | 85 | Male | 100 | 30 | 35 | 186 | 125 | Lateral | 2.0 | 6.4 | 99.0 |

BiV indicates bi-ventricular; LVIDd, left ventricular end diastolic diameter; EF, ejection fraction.
